# Congenital High Airway Obstructive Syndrome (CHAOS) Survival of a Newborn with Laryngeal Atresia

**DOI:** 10.3390/diagnostics13243658

**Published:** 2023-12-14

**Authors:** Carmen Heriseanu, Mihaela Bizubac, Loredana Draghia, Veronica Marcu, Dan Gheorghe, Catalin Cirstoveanu

**Affiliations:** 1 Neonatal Intensive Care Unit, “M.S. Curie” Children’s Hospital, Constantin Brâncoveanu Boulevard, No. 20, 4th District, 041451 Bucharest, Romania; mariana-carmen.iliescu@drd.umfcd.ro (C.H.); draghia_loredana@yahoo.com (L.D.); catalin.cirstoveanu@umfcd.ro (C.C.); 2 Faculty of Medicine, “Carol Davila” University of Medicine and Pharmacy, 020021 Bucharest, Romania; 3 Department of Neonatal Intensive Care, “Carol Davila” University of Medicine and Pharmacy, 020021 Bucharest, Romania; 4 Department of Radiology, “M.S. Curie” Children’s Hospital, Constantin Brâncoveanu Boulevard, No. 20, 4th District, 041451 Bucharest, Romania; veronicamarcu@yahoo.com; 5 Department of ENT, “Carol Davila” University of Medicine and Pharmacy, 020021 Bucharest, Romania; cristian.gheorghe@umfcd.ro

**Keywords:** CHAOS, laryngeal atresia, fetoscopy, tracheostomy, laryngotracheoplasty

## Abstract

Congenital high airway obstructive syndrome (CHAOS) is a rare congenital anomaly, frequently caused by laryngeal or tracheal atresia, tracheal stenosis, and obstructing laryngeal cysts. This is a congenital malformation, often fatal, with an unknown prevalence. Laryngeal atresia is the most frequent cause. We report a case of an intrauterine diagnosis of CHAOS and ascites in a 17-week fetus delivered at 38 weeks of gestation without other associated malformations. A fetoscopic procedure was performed at 22 weeks of gestation. An attempt was made to perforate the affected area to ensure pulmonary fluid circulation and the ascites’ resolution. After birth, a tracheostomy was performed. The patient was mechanically ventilated until 11 months of age, when she was discharged with no cerebral or other complications of immediate postnatal anoxia or episodes of respiratory arrest. A laryngotracheoplasty was performed at 2 years old, but decannulation was not possible due to certain complications. At 5 years old, a new surgical intervention was performed, which allowed decannulation 6 months later.

Introduction: CHAOS is a rare congenital anomaly, caused by laryngeal or tracheal atresia, tracheal stenosis, obstructing laryngeal cysts, and obstructing tumors of the oropharynx and the cervical region, or by compression from a double aortic arch [1]. This pathology was first described by Hedrick in the late 1900s [2], with a prevalence that is difficult to assess, mainly because the affected fetuses die in utero or at birth, and the anomaly can only be observed at postmortem examination [3,4]. Consequently, prenatal diagnosis is extremely important in the management of the disease. A theory regarding pathogenesis indicates the decrease in blood supply during embryogenesis, resulting in abnormal development of the trachea or larynx [5].

The anomaly is characterized by complete or incomplete airway obstruction. It is frequently caused by laryngeal atresia, but there may be other causes: tracheal or laryngeal agenesis, tracheal atresia, subglottic stenosis, or laryngeal cysts.

Prenatal diagnosis by means of ultrasound screening plays a crucial role in the evolution and prognosis of the condition, as it can reveal congenital high airway obstruction syndrome, which includes the fetus having hyperinflated, enlarged, and highly echogenic lungs, a dilated tracheobronchial tree, a flattened or inverted diaphragm, and ascites [6]. A fetal MRI exam can indicate the location and size of the obstruction, as well as the association with other anomalies. In undiagnosed patients who survive, respiration is possible through the existence of a tracheoesophageal fistula/the persistence of the pharyngotracheal duct [2,7,8].

The differential diagnosis considers Type 3 adenomatoid lung malformation, bronchial atresia, and airway obstruction from compression by an external mass (lymphatic malformation, cervical teratoma, vascular rings a like double aortic arch) [3,6]. 

CHAOS also associates with other anomalies, such as in the central nervous system (hydrocephaly, malformations of the aquaeductus), gastrointestinal system (esophageal atresia, tracheoesophageal fistula), urogenital system (hypoplasia of kidney, urethral atresia, vesicovaginal fistula), or skeletal system (varus deformity of feet, partial absence of cervical vertebrae, absence of radius, syndactyly) [9], as well as Fraser’s syndrome (urogenital defects, laryngeal atresia, syndactyly and cryptophthalmos) [10], cri-du-chat syndrome, short-rib polydactyly syndrome, and velocardiofacial syndrome [8,10,11]. 

The treatment of children with CHAOS can be divided into immediate and long-term care. Surviving patients are closely monitored, with an emergency tracheostomy performed immediately after birth, which seems to be the only chance of survival for the affected fetuses. The EXIT procedure (ex utero intrapartum treatment) is described as the only chance of survival for a patient with CHAOS. This consists of obtaining an intact airway for the baby before the fetomaternal circulation is stopped [12,13]. Airway reconstruction is delayed until the child is stabilized and allowed to grow [14]. 

The prognosis of larynx atresia is poor if not for an accurate and timely diagnosis, or when other severe anomalies are associated. Only very few cases of surviving newborns are reported in the literature. One of these is in good health at ten months of age, with no obvious neurological defects, in spite of a 15 min anoxia at birth [15,16].

Case Description: We report a case of an intrauterine diagnosis of CHAOS (congenital high airway obstructive syndrome) and ascites in a 17-week fetus delivered at 38 weeks of gestation without other associated malformations.

Our patient, ♀, conceived via in vitro fertilization and by parents with no concerning family history or consanguinity, was diagnosed at 17 weeks of gestation with CHAOS through ultrasound screening: hyperechoic lungs, much larger in volume, with a compressive effect on large vessels, and a squeezed heart (Figure 1a); the trachea was fully visualized up to the upper 1/3 of the fetal neck where stenosis occurred (pencil tip image) (Figure 1b); the diaphragm was flattened (Figure 1c); and ascites was present from the difficulty of venous return due to increased intrathoracic pressure (Figure 1d).

Considering the diagnosis and evolution of the disease, a fetoscopy was performed at 22 weeks of gestation, and a perforation (balloon dilation) of the affected area was attempted. The procedure had no adverse effects or complications for the mother or for the fetus. Subsequent ultrasounds revealed the spontaneous remission of ascites at 33 weeks of gestation.

The information brought by the ultrasound was supplemented with a fetal magnetic resonance imaging (MRI) exam at 30 weeks, which confirmed the diagnosis of laryngeal atresia and revealed the level of the obstruction. This exam showed the airway’s discontinuity, hyperechogenic lung, and flattened diaphragm Figure 2).

Despite the diagnosis, the couple decided to continue the pregnancy. The baby was delivered via cesarean section at 38 weeks of gestation, presenting with apnea and bradycardia. An orotracheal intubation attempt failed, and an emergency tracheostomy was performed. The newborn was transferred to our clinic after the first 3 h of neonatal life, in severe general condition, being mechanically ventilated on an intubation tube inserted in the tracheostomy and with inotropic support. An X-ray from admission showed bilaterally hyperinflated lungs with a flat diaphragm (Figure 3).

On the second day of life, a tracheostomy cannula was placed and a bronchoscopy was performed to establish the diagnosis: omega epiglottis, vocal cords, and laryngeal or tracheal lumen could not be visualized (Figure 4).

In the first months of life, the patient presented episodes of respiratory arrest with air trapping, but was responsive to the squeezing of the thorax and to the change in the ventilation mode in SIMV (synchronized intermittent mandatory ventilation), prior to which the CMV (continuous mandatory ventilation), the HFOV (high-frequency oscillatory ventilation), and negative pressure ventilation modes had been unsuccessfully tried.

The computed tomography (CT) scan revealed a c.a. 10 mm discontinuity in the upper airways and abnormal bronchial caliber, with ventilation disorders consistent with the respiratory symptomatology of the patient (Figure 5 and Figure 6).

A direct laryngoscopy was performed at the age of 9 months, revealing a complete obstruction of the glottis, with a very small fistula between the arytenoid cartilages (Figure 7). 

In time, an improvement in bronchial diameter was observed with weight gain. The baby required long-term ventilatory support, slowly decreasing the ventilation parameters, and at 11 months, she was able to breathe room air spontaneously through a tracheotomy cannula. 

A brain MRI examination performed before discharge to assess the presence of neurological sequelae did not reveal abnormalities at this level. However, the presence of a formation suggestive of presacral teratoma was observed (an MRI of the spine was performed because the physical examination revealed the presence of a presacral fossa, and the neurosurgical exam recommended it) (Figure 8b).

Following a 1-year hospitalization period, she was discharged in good general condition, without respiratory support, and with oral feeding. A slight delay in neuromotor development was observed but later recovered. 

A laryngotracheoplasty was attempted at 25 months old in our country. A spheric cricoid cartilage was found instead of a normal one, and no intra-laryngeal space was present. The very small fistula observed during the laryngoscopy ran posteriorly to the cricoid cartilage. Laryngo-tracheal reconstruction surgery was performed using posterior and anterior augmentation, with costal cartilage grafts, after extensive drilling of the primitive cricoid (Figure 9).

Due to the poor postoperative sedation of the child and thin cricoid remains following the drilling, the posterior graft was expelled during a coughing fit. An early postoperative endoscopy showed the persistence of a small subglottic space (unhealed at the time) and posterior glottic stenosis (due to insufficient local mucosa) between the arytenoid cartilages (Figure 10). 

At 2.5 years of age, the open posterior augmentation laryngotracheoplasty was repeated, followed by stenting with a Montgomery T-tube, due to multiple cartilaginous defects observed at the suprastomal trachea (Figure 11). 

After stent removal, the patient developed a new asymmetrical gloto-subglottic stenosis, a broadened anterior commissure (due to anterior cartilage grafting), and some left vocal cord movement impairment (see Figure 12). We did not perform any other surgery from that moment on, considering the age-related changes in the larynx to be more important for further operative management. Thus, at the age of 5, a new surgical intervention (anterior laryngoplasty with costal cartilage, partial left arytenoidectomy, and aryepiglottic fold resection) was considered appropriate and was performed at a hospital abroad with successful decannulation 6 months later.

Discussion: Congenital high airway obstruction syndrome (CHAOS) is a rare and usually lethal disease. The malformation is caused by the nondevelopment of the sixth branchial arch during normal embryological development. Smith and Bain have classified laryngeal atresia into three types: type 1—complete atresia of the larynx with midline fusion of the arytenoid cartilages and intrinsic muscles; type 2—infraglottic obstruction that is characterized by dome-shaped cricoid cartilage obstructing the lumen; and type 3—occlusion of the anterior fibrous membrane and fusion of the arytenoid cartilages at the level of the vocal processes [15,17]. Many causes have been described, including laryngeal or tracheal webs, laryngeal cysts, tracheal atresia, subglottic stenosis or atresia, and laryngeal or tracheal agenesis. Laryngeal atresia seems to be the most common cause [18], as was the case with our patient.

Laryngeal atresia may be associated with other structural and genetic defects [19], such as a left persistent superior vena cava, a single umbilical artery, abnormal fingers and toes, esophageal atresia, and renal agenesis [20,21]. Partial trisomy 9 and 16, chromosome 5p deletion, and 11q11.2 deletion are also associated [22,23,24]. Our patient did not present with any of the defects described in the literature, but an MRI exam performed at the age of 1 revealed the presence of a presacral teratoma.

Our patient’s diagnosis was made using the main diagnostic method, an ultrasound screening at 17 weeks of gestation, which revealed the presence of all specific signs: large hyperechoic lungs and a squeezed heart, with tracheal obstruction, a flattened diaphragm, and ascites (without signs of heart failure). A fetal MRI examination was also performed at 30 weeks, which confirmed the diagnosis of laryngeal atresia and indicated the level of the obstruction.

In most cases described in the medical literature, following the initial diagnosis of CHAOS, the parents are informed about the risks and the effects of the condition, and genetic counseling is recommended, resulting in them frequently opting for terminating the pregnancy. In our case, the parents decided to continue the pregnancy.

This pathology involves tracheobronchial tree obstruction, which causes perturbations in the pulmonary fluid circulation with an increase in intratracheal pressure, resulting in lung enlargement, which, in turn, leads to the compression of the heart and the great vessels with a decrease in cardiac output and venous return, causing dysfunctions at the cardiovascular system level and the appearance of ascites. For this reason, in the intrauterine management of CHAOS, decompression of the airways via a fetoscopy was attempted to ensure lung development and decrease the risk of pre- and postnatal death [7,25,26]. Reverse fetal hydrops were observed, with a favorable impact. A fetoscopy was also chosen in the case of our patient at 22 weeks of gestation. An attempt was made to perforate the obstructive area to lower the intrathoracic pressure by removing the pulmonary fluid. As a result, a decrease in the level of ascites was observed up to total disappearance in week 33. Postnatal, perhaps due to ascites, at the clinical examination, she presented a globular abdomen, with a visible intestinal contour and hypotonia of the abdominal muscles.

The postnatal management of newborns with CHAOS is difficult, and the prognosis is poor. Without intervention, the postnatal mortality rate is 100% [5]. In patients with an antenatal diagnosis whose parents decided to continue the pregnancy, the EXIT procedure (ex utero intrapartum treatment) is performed. This is widely viewed as the best course of action to ensure the survival of these patients. The main goal of the procedure is to establish a safe airway for the child before maternal–fetal circulation is interrupted [12,27,28]. Once the cephalic extremity is exposed, a laryngo/tracheoscopy can be performed initially, as in case of obstructions due to the existence of a membrane, that can be disrupted followed by endotracheal intubation. But if an atretic segment is observed, a tracheostomy is performed, thus increasing the chances of survival and a better prognosis. Following this diagnosis, a multidisciplinary team was formed (obstetrician, anesthesiologist, pediatric surgeon/otolaryngologist, neonatologist), and at 38 weeks of gestation, the fetus was extracted via cesarean section. Although the diagnosis was antenatal, the EXIT procedure was not performed, hoping for a favorable fetoscopy result. Instead, an oro-tracheal intubation was attempted, followed by a tracheostomy.

With regard to our patient’s prognosis, all criteria for an unfavorable evolution were met from the beginning: a flattened diaphragm with hyperinflation of the lungs and significant modification of the bronchial caliber. Although a tracheostomy was performed immediately after birth, spontaneous breathing could not be achieved due to abnormal bronchopulmonary development induced by the respiratory tree obstruction. This entailed the need for prolonged mechanical ventilation (different types of mechanical ventilation with positive and negative pressure), followed by the development of respiratory complications with episodes of air trapping and respiratory arrest that responded well to disconnecting the ventilator for a few seconds and squeezing the thorax. In time, the symptomatology improved and the child could be disconnected for short periods of time from the ventilator until the age of 11 months, when weaning was possible.

The patient was discharged from the hospital at the age of 11 months, with no cerebral or other complications resulting from postnatal anoxia or episodes of respiratory arrest. The physical examinations, ultrasound scans, CT, or MRI scans showed no pathological signs.

As far as surgical treatment was concerned, several methods of tracheal replacement have been proposed, including the use of allografts, prosthetic materials, autologous tissue, or a combination of these materials [29]. Autologous costal cartilaginous tissue was eventually used for the surgery performed in our country when the patient was 25 months old. Due to complications, the suppression of the tracheostomy tube was not possible at that time. But, at the age of 5, a new surgical intervention was performed (anterior laryngoplasty with costal cartilage, partial left arytenoidectomy, and aryepiglottic fold resection) at a hospital abroad, with successful decannulation 6 months later.

It is very important to diagnose the atresia of the larynx antenatally using ultrasound screening because advanced techniques of fetal surgery can correct certain defects through fetal tracheoplasty or total resection with anastomosis [30], and an antenatal diagnosis significantly increases the chances of postnatal survival.

Conclusions: Laryngeal atresia is generally fatal. Early intrauterine diagnosis (via ultrasound, MRI exam) of fetuses with laryngeal atresia is essential to establish the therapeutic conduct and increase the chances of survival. Decompression of the airways via a fetoscopy ensures lung development and decrease the risk of pre- and postnatal death.

In this case, because the fetal ascites disappeared, the authors were convinced that fetoscopy is a success in permeabilizing the airways. For this reason, the EXIT maneuver was not performed at birth. Unfortunately, this hypothesis was untrue, and the baby required an emergency tracheostomy.

The postnatal management of newborns with CHAOS is difficult, and the prognosis is often unfavorable. The care of these children requires the existence of a multidisciplinary team.

Newborns who survive require prolonged hospitalization, with prolonged mechanical ventilation secondary to their abnormal bronchopulmonary development.

With the evolution of surgical procedures, these patients may benefit from laryngeal reconstruction and tracheostomy closure.

## Figures and Tables

**Figure 1:**
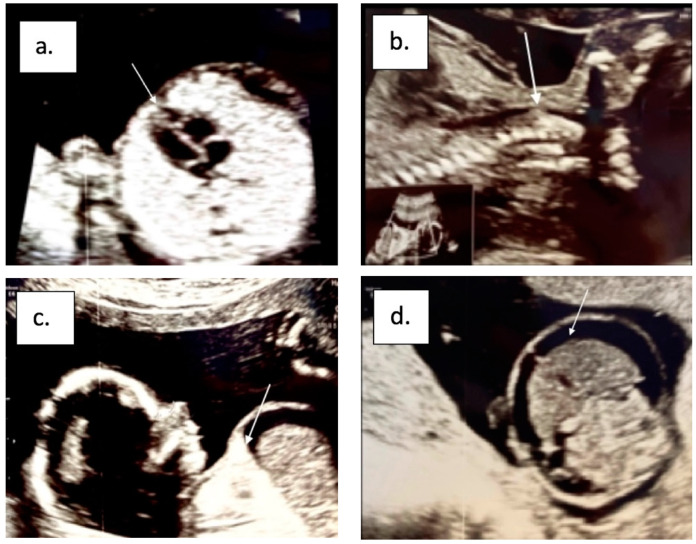
Antenatal sonography showing (**a**) large hyperechoic lungs and squeezed heart, (**b**) tracheal obstruction, (**c**) flattened diaphragm, and (**d**) ascites.

**Figure 2:**
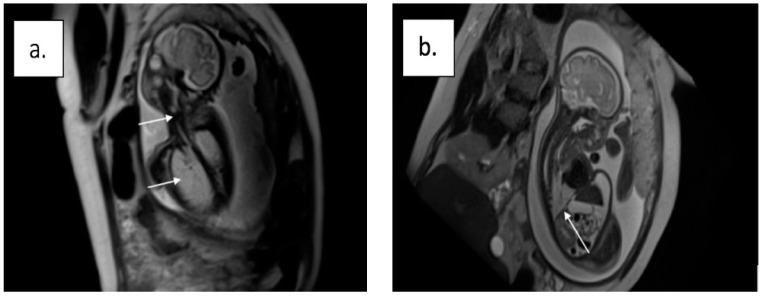
Fetal magnetic resonance image at 30 weeks of gestation: (**a**) airway discontinuity, hyperechogenic lung, and (**b**) flattened diaphragm.

**Figure 3:**
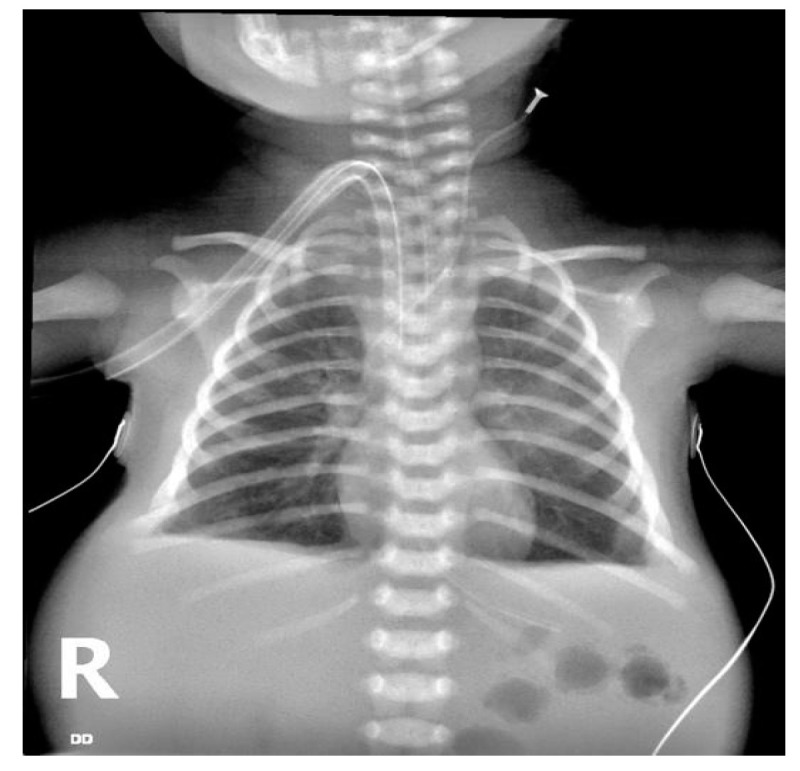
Chest X-ray at birth showing hyperinflated lungs, flattened diaphragm, and enlarged abdomen secondary ascites.

**Figure 4:**
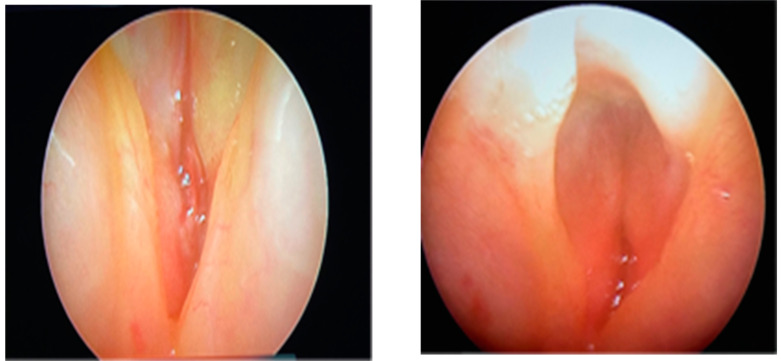
Rigid laryngeal endoscopy: omega epiglottis, vocal cords, and laryngeal or tracheal lumen cannot be visualized.

**Figure 5:**
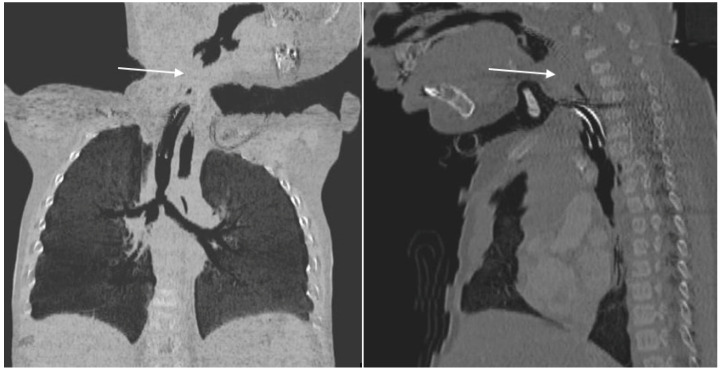
Discontinuity above the tracheostomy cannula.

**Figure 6:**
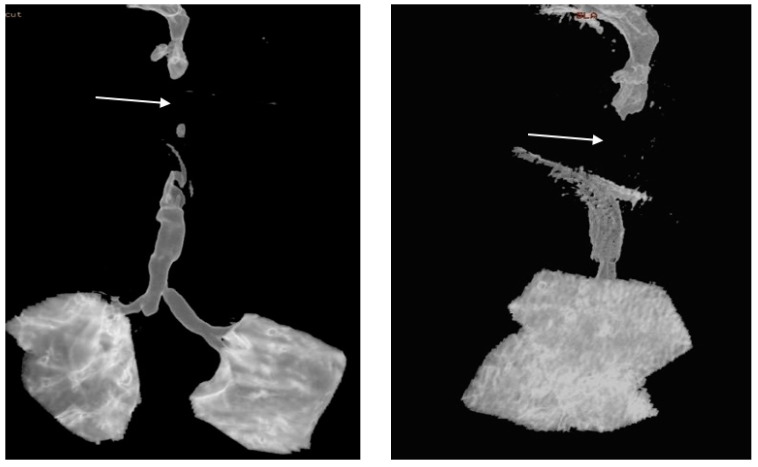
3D airway reformatting.

**Figure 7:**
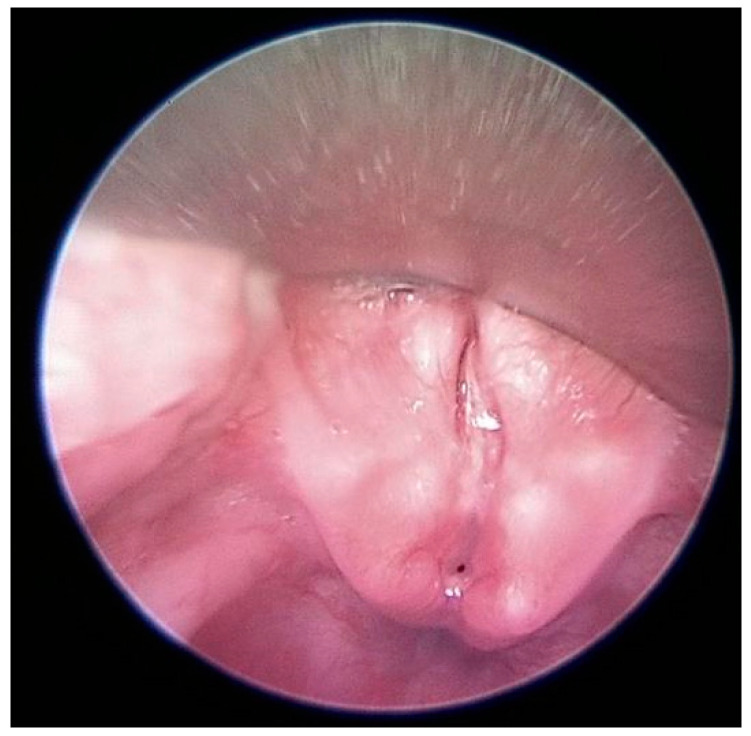
Direct laryngoscopy at 9 months old.

**Figure 8:**
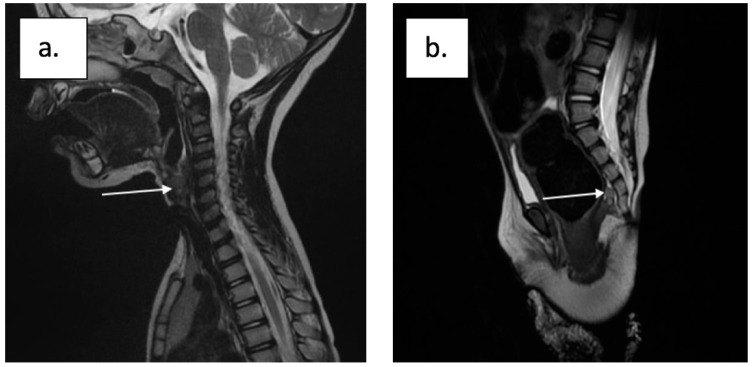
Magnetic resonance image showing (**a**) airway obstruction, (**b**) coccyx agenesis, and presacral teratoma.

**Figure 9:**
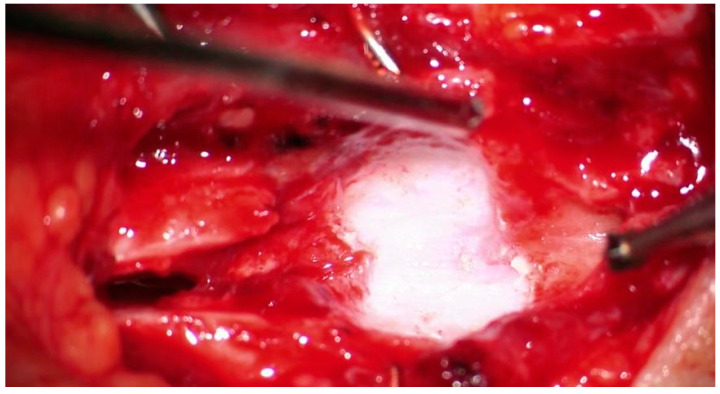
Cricoid cartilage after drilling.

**Figure 10:**
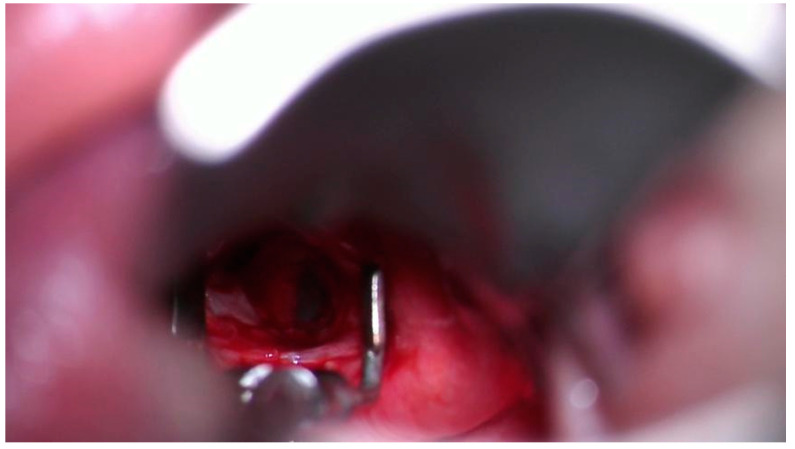
Subglottic narrowing of posterior glottic stenosis.

**Figure 11:**
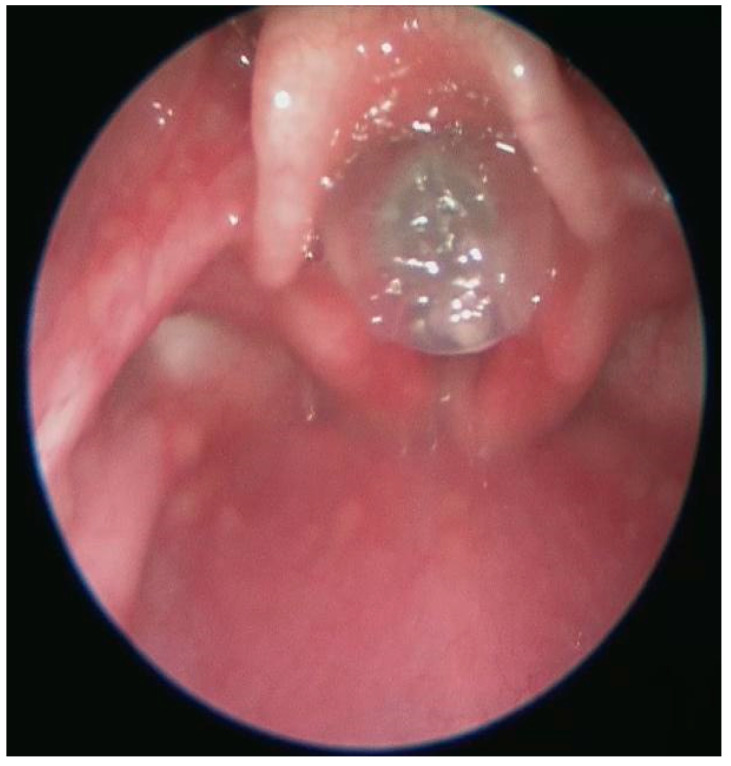
Montgomery T-tube in larynx and trachea (with capped upper extremity).

**Figure 12:**
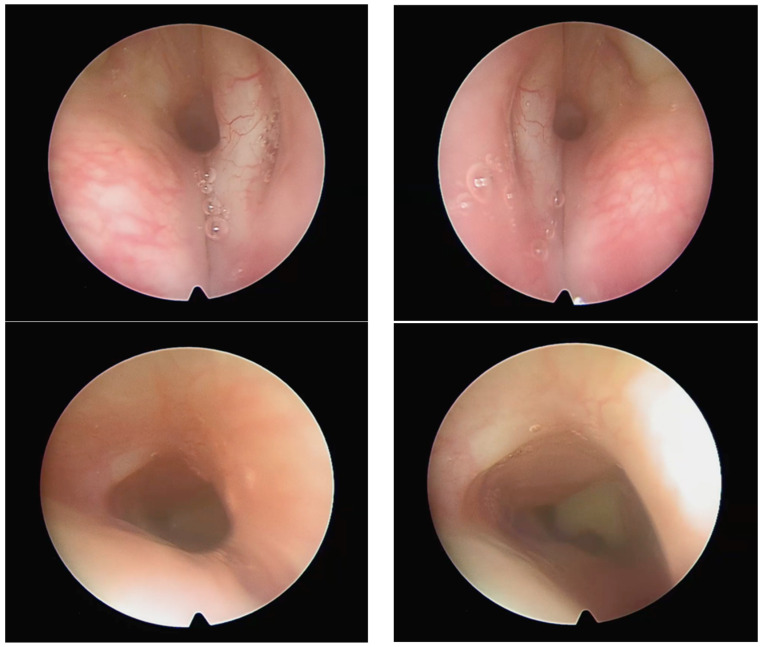
Images after 18 months post laryngo-tracheal reconstruction.

## Data Availability

Data are available on request from the corresponding author.
